# Development of Biotinylated Liposomes Encapsulating Metformin for Therapeutic Targeting of Inflammation-Based Diseases

**DOI:** 10.3390/pharmaceutics16020235

**Published:** 2024-02-05

**Authors:** Giorgia Ailuno, Sara Baldassari, Alice Balboni, Sara Pastorino, Guendalina Zuccari, Katia Cortese, Federica Barbieri, Giuliana Drava, Tullio Florio, Gabriele Caviglioli

**Affiliations:** 1 Department of Pharmacy, University of Genoa, Viale Cembrano 4, 16148 Genova, Italy; sara.baldassari@unige.it (S.B.); alice.balboni@edu.unige.it (A.B.); guendalina.zuccari@unige.it (G.Z.); giuliana.drava@unige.it (G.D.); gabriele.caviglioli@unige.it (G.C.); 2 Territorial Pharmacy of Azienda Sociosanitaria Ligure 2, Via Carlo Collodi 13, 17100 Savona, Italy; sa.pastorino@asl2.liguria.it; 3 Department of Experimental Medicine, University of Genoa, Via Antonio de Toni 14, 16132 Genova, Italy; cortesek@unige.it; 4 Department of Internal Medicine, University of Genoa, Viale Benedetto XV 2, 16132 Genova, Italy; federica.barbieri@unige.it (F.B.); tullio.florio@unige.it (T.F.); 5 IRCCS Ospedale Policlinico San Martino, Largo Rosanna Benzi 10, 16132 Genova, Italy

**Keywords:** liposomes, VCAM-1, targeting, inflammation, cancer, avidin–biotin complex, metformin hydrochloride

## Abstract

Inflammation is a physiological response to a damaging stimulus but sometimes can be the cause of the onset of neurodegenerative diseases, atherosclerosis, and cancer. These pathologies are characterized by the overexpression of inflammatory markers like endothelial adhesion molecules, such as Vascular Cell Adhesion Molecule-1 (VCAM-1). In the present work, the development of liposomes for therapeutic targeted delivery to inflamed endothelia is described. The idea is to exploit a three-step pretargeting system based on the biotin–avidin high-affinity interaction: the first step involves a previously described biotin derivative bearing a VCAM-1 binding peptide; in the second step, the avidin derivative NeutrAvidin^TM^, which strongly binds to the biotin moiety, is injected; the final step is the administration of biotinylated liposomes that would bind to Neutravidin^TM^ immobilized onto VCAM-1 overexpressing endothelium. Stealth biotinylated liposomes, prepared via the thin film hydration method followed by extrusion and purification via size exclusion chromatography, have been thoroughly characterized for their chemico-physical and morphological features and loaded with metformin hydrochloride, a potential anti-inflammatory agent. The three-step system, tested in vitro on different cell lines via confocal microscopy, FACS analysis and metformin uptake, has proved its suitability for therapeutic applications.

## 1. Introduction

It has been demonstrated that inflammation, besides resulting from a physiologic reaction to potentially noxious stimuli, such as exposure to pathogens or tissue damages, is also involved in the onset and progression of some widespread pathologies like cancer [1,2], atherosclerosis [3,4], rheumatoid arthritis [5], cardiac allograft rejection [6], and neurodegenerative [7] and pulmonary [8,9] diseases.

The inflammatory response involves a number of mediators (such as interleukins, prostaglandins, leukotrienes, peptides like bradykinin and proteins like the platelet-activating factor) that induce the sequence of responses promoting the activation of the endothelium of the blood capillaries in the interested area, and the stimulation of blood cells such as monocytes [10]. Within this process, endothelial cells lose their glycocalyx [11] and overexpress some adhesion proteins exploited by monocytes for their migration. Different types of adhesion molecules are expressed by endotheliocytes, like selectins, integrins, Vascular Cell Adhesion Molecule-1 (VCAM-1) and Intercellular Adhesion Molecule-1 (ICAM-1) [12].

In recent years, many attempts have been made to exploit the endothelial overexpression of adhesion molecules for the diagnosis and therapy of inflammatory-based diseases due to their easy accessibility on the vascular surface [13]. In particular, VCAM-1 has emerged as a favorable target, as it is scarcely expressed in healthy tissues, while it is precociously and conspicuously exposed by the cells of the inflamed tissues [14,15]. A wide variety of strategies has been proposed for VCAM-1 targeting, for example, based on the use of antibodies and their fragments or peptides [12].

**Figure 1:**
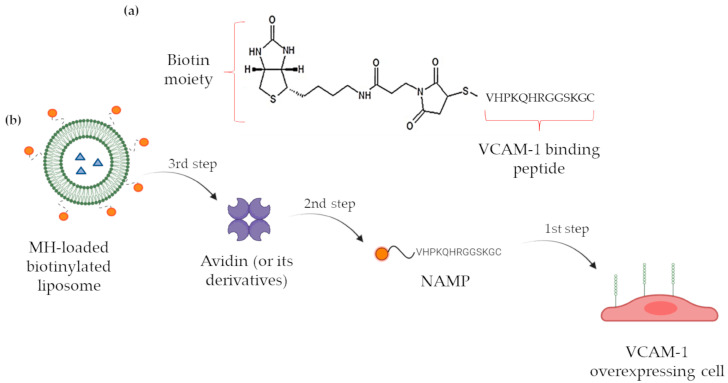
(**a**) NAMP structure; (**b**) schematic representation of the three-step pretargeting system. Created with Biorender.com, accessed on 19 December 2023.

In a previous work [16], our research group disclosed a novel VCAM-1 targeting PET radiotracer, based on the VHPKQHRGGSKGC VCAM-1 binding peptide, containing the VHPKQHR amino acid sequence, whose VCAM-1 affinity has been deeply investigated [17,18,19]. The newly synthesized molecule, named NAMP (Figure 1a), is a biotin derivative conceived to be used in a three-step pretargeting system exploiting the biotin/avidin high-affinity complex for the visualization of VCAM-1 overexpressing endothelial regions; after the administration of NAMP, selectively binding to VCAM-1, avidin or avidin-derived molecules such as Neutravidin^TM^, aimed at binding NAMP biotin moiety, would be administered; finally, in our previous work, we conceived a third step consisting of the administration of a double-chelating biotinylated radiotracer as a diagnostic PET imaging agent.

However, the three-step pretargeting system might also be exploited for therapeutic applications, replacing the biotinylated radiotracer with a biotinylated drug delivery vehicle, which might even include an imaging agent, turning into a flexible theranostic system. Among the possible nanocarriers, we decided to exploit liposomes since they have already demonstrated their effectiveness as drug vehicles in a wide variety of pathologies [20,21] and their applicability in clinics [22,23]. Moreover, among the possible effective cargoes, metformin hydrochloride (MH) might be included. Indeed, besides the acknowledged antidiabetic activity, metformin has shown the property of reducing the inflammation phenomena in endotheliocytes and conditions such as death caused by cardiovascular diseases, stroke, and thrombotic risk factors [24,25,26]. The administration of MH has proved to reduce the levels of soluble adhesion molecules [15,27,28], and this activity is apparently independent of the hypoglycemic effect of the drug, but rather ascribable to the diminished production of inflammation markers such as nuclear factor kappa B (NF-κB) [25]. In addition, many studies have reported the successful application of MH in the treatment of various forms of cancer to which diabetic patients are unfortunately prone: the evidence suggests that those patients receiving MH benefit from reduced cancer risk and mortality [29,30,31,32].

On the basis of this consideration and of our background in the development of analytical methods for metformin dosing [33,34], we have formulated and developed biotinylated liposomes, which have been loaded with MH and successfully tested for their targeting abilities in vitro, thus demonstrating the feasibility of the pretargeting nanosystem as an innovative and versatile theranostic tool [35] (Figure 1b).

## 2. Materials and Methods

### 2.1. Chemicals

1,2-dioleoyl-sn-glycero-3-phosphocholine (DOPC), cholesterol ≥ 99%, dimethyldioctadecylammonium bromide ≥ 98% (DMDAB), HEPES sodium salt, potassium phosphate monobasic, praseodymium nitrate hexahydrate, sodium chloride, sodium phosphate dibasic diihydrate, HABA (4′-hydroxyazobenzene-2-carboxylic acid), and *N*-methyl-bis(trifluoroacetamide) were supplied by Sigma-Aldrich Inc. (St. Louis, MO, USA); 1,2-distearoyl-sn-glycero-3-phosphoethanolamine-*N*-[biotinyl(polyethylene glycol)-2000] (ammonium salt) (DSPE-PEG_2000_-biotin), and 1,2-distearoyl-*sn*-glycero-3-phosphocholine (DSPC) were obtained from Avanti Polar Lipids Inc. (Alabaster, AL, USA); 1,2-distearoyl-sn-glycero-3-phosphoethanolamine-*N*-[methoxy(polyethylene glycol)-2000] (ammonium salt) (DSPE-PEG_2000_) was provided by Nanocs Inc. (New York, NY, USA); [^2^H_6_]-metformin hydrochloride was purchased from Metapharmaceutical (Barcelona, Spain); CellTracker™ CM-DiI Dye was supplied by Invitrogen Co. (Waltham, MA, USA); metformin hydrochloride was obtained from Metapharmaceutical (Barcelona, Spain); reagent-grade cyclohexane, aluminum sheet silica gel 60 F254 plates, and 100 kDa MWCO Amicon ultra centrifugal filters were obtained from Merck (Darmstadt, Germany); chloroform stabilized with about 0.6% ethanol, diethyl ether, and methanol were supplied by VWR (Radnor, PA, USA); polycarbonate membranes with 400, 200 and 100 nm pore diameters were acquired from Avestin Europe GmbH (Mannheim, Germany). The water used was purified with a Milli-Q system (Millipore, Burlington, MA, USA). All chemicals were used as received without further purification.

### 2.2. Liposome Formulation

Two different formulations of liposomes, anionic (F1) and cationic (F2), were formulated by applying the thin film hydration method, starting from the lipid mixtures detailed in Table 1.

Appropriate aliquots of stock solutions obtained by dissolving appropriate amounts of lipids in chloroform were mixed and vacuum-dried. The obtained lipid film was hydrated with Milli-Q water or phosphate-buffered saline (pH 7.2) and added to CaCl_2_ · 2H_2_O and MgCl_2_ · 6H_2_O (to a final concentration of 0.9 mM and 0.5 mM, respectively) or HEPES buffer 10 mM, 150 mM NaCl (pH 7.4). The different hydration media were selected depending on the tests to be successively performed. The F1 formulation was hydrated at 58 ± 1 °C for 2 h, while the F2 formulation was hydrated at 46 ± 1 °C for 2 h. The extrusion of the obtained dispersions was carried out through the use of polycarbonate filters of 400, 200 and 100 nm pore size with a Liposofast pneumatic extruder (Avestin, Mannheim, Germany), which was immersed in a water bath at 58 °C or 46 °C for the F1 and F2 formulations, respectively, under approx. 4 bars of pressure. The extrusion sequence was optimized as follows: 9 times through the 400 nm pore size filter, 11 times through the 200 nm pore size filter, and 13 times through the 100 nm pore size filter.

For the in vitro studies, the liposomes were labeled with CM-DiI fluorescent dye by adding a 0.1 μg/μL ethanolic solution of the dye to the lipid solution before film formation.

### 2.3. MH Loading

F1 liposomes encapsulating MH were prepared via three different procedures based on the thin film hydration method.

Formulation F1a-MH

MH was dissolved in Milli-Q water to a concentration of 10 mg/mL; 2 mL of the solution was employed to hydrate the thin film obtained, as described in Section 2.2, from a 20 µmol total lipid mixture in a water bath at a temperature of about 58 °C for 2 h. The resulting dispersion was extruded, as described in Section 2.2.

Formulation F1b-MH

MH was dissolved in methanol to a concentration of 10 mg/mL. After the lipid thin film preparation, 2 mL of the methanolic solution was added, and the solvent was evaporated under reduced pressure. The film was hydrated with 2 mL of Milli-Q water at about 58 °C for 2 h. The resulting dispersion was extruded, as described in Section 2.2.

Formulation F1c-MH

MH was dissolved in methanol to a concentration of 10 mg/mL. Further, 2 mL of this methanolic solution was added to appropriate volumes of lipid chloroformic stock solutions in order to achieve the mol% reported in Table 1. After the mixture was vacuum-dried, 2 mL of Milli-Q water was added for hydration under rotation at about 58 °C for 2 h.

The resulting dispersion was extruded, as described in Section 2.2.

### 2.4. Liposomes Purification

Both unloaded and drug-loaded liposomal suspensions were purified through the use of semipreparative HPLC using a Waters 1525 binary pump system (Waters Co., Milford, MA, USA) through size exclusion chromatography (SEC), employing a TSKgel G6000pw column (internal diameter 0.75 cm; length 30 cm) (Tosoh Bioscience, Tokyo, Japan) thermostated at 30 °C for the whole duration of the purification, with injection volumes of 400 μL. Isocratic elution was carried out with a flux of 1 mL/min of Milli-Q water. For detection, a Waters 2414 refractive index (RI) detector was employed, with its sensitivity set to 16 and a time constant of 1, thermostating the detection cell at 30 °C; in addition, a Waters 2998 photodiode array (PDA) detector was employed, monitoring 210, 254, and 280 nm wavelengths, plus λ = 570 nm as the analytical wavelength when purifying CM-DiI-labeled liposomes, and λ = 233 nm as the analytical wavelength when purifying MH-loaded liposomes. HPLC data were analyzed using Waters Empower^TM^ 3 software version 18.4.0.0.0.

After purification, the isolated liposome dispersions were concentrated via ultrafiltration using Amicon 100 kDa centrifugal devices in order to achieve a final theoretical lipid concentration suitable for the tests to be successively performed.

The liposomal batches used for cholesterol content and drug loading determination were directly lyophilized after SEC purification.

### 2.5. Liposome Characterization

#### 2.5.1. Size, Zeta Potential, Concentration, and Stability

For size and zeta potential measurements, the liposome formulations were analyzed using a Zetasizer HSa 3000 analyzer (Malvern Ltd., Malvern, UK) with a He/Ne lamp (λ = 633 nm). To perform the measurements, the liposomes were diluted with 0.01 N NaCl aqueous solution. The mean hydrodynamic diameter was measured via dynamic light scattering (DLS) analysis and was the result of three measurements (10 sub runs each). The signal is first acquired by the software in the non-negative least squares mode and then re-elaborated through the CONTIN algorithm. The zeta potential was measured through the use of an electrophoretic mobility assessment by injecting the sample into the measurement cell.

To determine the liposome concentration, nanoparticle tracking analysis (NTA) was performed by using a NanoSight NS300 (Malvern Ltd, Malvern, UK). For this kind of analysis, purified liposomal suspensions were diluted to 1:10,000 in Milli-Q water.

The physical stability of the dispersions was evaluated by monitoring the size and zeta potential over two weeks of storage at a temperature of 4 °C. For F1 liposomes, the stability study was continued for one year.

#### 2.5.2. Qualitative Determination of the Presence of Biotinylated Lipid

In order to confirm liposome–NeutrAvidin^TM^ binding, an assay based on biotin displacement on the 4-hydroxyazobenzene-2-carboxylic acid (HABA)–NeutrAvidin^TM^ complex was performed. HABA was dissolved in Milli-Q water to a concentration of 0.22 mg/mL, and then 50 μL of this aqueous solution was mixed with 1.5 mL of a NeutrAvidin^TM^ solution in Milli-Q water (concentration 0.5 mg/mL): the HABA–NeutrAvidin^TM^ complex formed, with all NeutrAvidin^TM^ binding sites saturated, and its UV absorbance at 500 nm was measured. Then, 0.5 mL of the liposome suspension was mixed with 0.5 mL of the HABA–NeutrAvidin^TM^ complex solution, and the displacement was assessed on the basis of the reduced UV absorbance at 500 nm.

#### 2.5.3. Transmission Electron Microscopy

After HPLC purification performed as described in Section 2.2, batches of liposome formulations were concentrated via filtration in a Beckman Avanti J-30I high-performance centrifuge (Beckman Coulter Life Sciences, Indianapolis, IN, USA) using Amicon 100 kDa centrifugal devices (1500 rpm, 20 min, 10 °C) and resuspended in PBS to obtain a final lipid concentration of approx. 20 mM. The liposomes were observed using a HT7800 transmission electron microscope (Hitachi, Tokyo, Japan) with a Megaview 3 digital camera and Radius software version 2.2. Negative staining was carried out as follows: 20 μL of 2% paraformaldehyde in 0.1 M phosphate buffer (pH 7.4) was added to an equal volume of liposomes in PBS; then, the liposomes were adsorbed to formvar–carbon-coated copper grids, which were floated on 5 μL drops on parafilm for 20 min. The grids were then rinsed with PBS and stained with 2% uranyl acetate for 5 min at room temperature. For improved preservation, stained grids were embedded in 2.5% methylcellulose and finally air-dried before examination.

#### 2.5.4. ^31^P-NMR for Lamellarity Determination

The splitting and shifting of the ^31^P NMR spectra of the liposomes were analyzed for the determination of their lamellarity using a 400 MHz-ECZ400R/S3 spectrometer (JEOL, Tokyo, Japan). A 25 mM aqueous solution of the shift reagent Pr(NO)_3_ ∙ 6 H_2_O was used. The NMR sample was prepared as follows: 400 μL of the liposome suspension (suitably concentrated to about 32.5 mM lipid concentration) was mixed with 320 μL of Pr(NO)_3_ ∙ 6 H_2_O solution and 80 μL of deuterated water. The control sample, prepared without the shift reagent, was obtained by adding 320 μL of Milli-Q water in place of the shift reagent solution. The measurements were carried out at 27 °C, acquiring 3000 scans, with a delay time of 5 s. During the complete measurement, broad-band ^1^H decoupling was applied.

Liposome lamellarity was calculated by applying the following equation (Equation (1)):

(1) $$\mathrm{Lamellarity}=\frac{\mathrm{Sum}\;\mathrm{of}\;\mathrm{the}\;\mathrm{areas}\;\mathrm{of}\;\mathrm{the}\;\mathrm{two}\;\mathrm{NMR}\;\mathrm{peaks}}{2\times \mathrm{area}\;\mathrm{of}\;\mathrm{shifted}\;\mathrm{peak}}$$

In the equation, the sum of the areas of the two peaks present in the spectra after the addition of the shift reagent is divided by twice the area of the shifted peak (i.e., the left-shifted peak appeared only when the shifting reagent is added).

#### 2.5.5. Cholesterol Content Determination

The cholesterol content in the liposomes was determined via the TLC method, employing TLC silica gel 60 F254 aluminum sheets with a layer thickness of 0.2 mm, without thermal activation. Thin bands of the sample and standard solutions were deposited using a semi-automatic applicator Linomat 5 (CAMAG, Muttenz, Switzerland) with the following settings: 6 mm band length, distances from the left plate and lower plate edge of 10 mm and 20 mm, respectively, and the distance between the adjacent tracks of 15 mm; 20 μL of each sample was applied under N_2_ flux at a speed of 250 nL/s.

Liposome samples were prepared by dissolving accurately weighed lyophilized liposome powder in chloroform.

Chromatography was carried out in a 20 cm × 10 cm twin trough chamber (CAMAG, Muttenz, Switzerland) using a mobile phase composed of cyclohexane/diethyl ether 3:2.5 (*v*/*v*) up to a migration distance of 95 mm (from the lower plate edge); finally, the plate was dried at 40 °C in a ventilated oven for 10 min.

The detection was carried out by monitoring the UV absorbance at 200 nm using a TLC Scanner 3 (CAMAG, Muttenz, Switzerland) with a scanning speed of 10 mm/s, a slit dimension of 5 mm × 0.45 mm, and a data resolution of 100 μm/step. The winCats 1.4.1 Planar Chromatography Manager software (CAMAG, Muttenz, Switzerland) controlled all instruments.

The cholesterol content in the liposomal formulations was quantified against a standard calibration curve (linearity range from 0.2 to 2 mg/mL, corresponding to 4 to 40 μg) and expressed as *w*/*w* percentage.

#### 2.5.6. MH Loading Determination

A TLC method was developed for the determination of the amount of MH loaded into liposomes, employing aluminum TLC Silica Gel 60 sheets without fluorescent indicator. The sample and standard solutions were deposited in bands, as described in Section 2.5.5.

MH-loaded liposome sample solutions were prepared by dissolving accurately weighed amounts of lyophilized liposome powder in methanol, while standard solutions were obtained by dissolving accurately weighed amounts of MH or mixtures of MH and lipids in methanol (mixed at the same theoretical lipid ratio composing the liposomal formulation).

Ammonium sulfate (0.025 M)/acetonitrile 7:3 (*v*/*v*) was used as the mobile phase, and chromatography was performed as described in Section 2.5.5. Finally, the plate was dried at 30 °C in a ventilated oven for 15 min.

Detection, performed by applying the settings reported in Section 2.5.5, consisted of measuring UV absorbance at 233 nm, and MH was quantified against a standard calibration curve, which showed linearity in the range from 25 to 100 µg/mL, corresponding to 0.5 to 2 μg MH.

The drug loading was calculated by applying the following equation (Equation (2)):

(2) $$\mathrm{Drug}\;\mathrm{loading}=\frac{\mathrm{Weight}\;\mathrm{of}\;\mathrm{MH}\;\mathrm{found}\;\mathrm{in}\;\mathrm{liposomes}}{\mathrm{Weight}\;\mathrm{of}\;\mathrm{lyophilized}\;\mathrm{formulation}}\times 100$$

#### 2.5.7. Cell Cultures

In vitro tests of the three-step pretargeting procedure were conducted on two different cell cultures: human glioblastoma cells, characterized by VCAM-1 overexpression, and human umbilical vein endothelial cells (HUVECs).

Human glioblastoma cells were obtained from postsurgical specimens of patients who underwent surgery at Ospedale Policlinico San Martino in Genoa, with informed consent obtained using the guidelines approved by the Institutional Ethical Committee (CER Liguria register number 360/2019). These cells, after transfection with a retrovirus expressing a green fluorescent protein (GFP), were cultured in an EndoGRO-LS Complete Culture Media Kit, and finally seeded on Matrigel-coated plates. For confocal microscopy observations, the cells were plated on microscopy dishes with a coverslip.

HUVECs were purchased from PromoCell (Heidelberg, Germany) and cultured in Endothelial Cell Growth Medium (PromoCell, Heidelberg, Germany). For fluorescence-activated cell sorting (FACS) experiments, a CytoFLEX S Flow Cytometer (Beckman Coulter Life Sciences, Indianapolis, IN, USA) was used. The cells were stimulated with TNF-α (10 ng/mL for 4 h), and then, after detachment from the plate using a scraper, the cells were transferred to Eppendorf tubes (approximately 10^5^ cells per tube). The data were analyzed by using FlowJo software X version 10.9 (Becton Dickinson, Franklin Lakes, NJ, USA), with standard gating to eliminate aggregates and dead cells.

#### 2.5.8. In Vitro Tests

By applying the three-step protocol, the two liposomal formulations were tested in vitro on human glioblastoma cells, with a final evaluation performed using confocal microscopy. Prior to the experiments, the cells were fixed with 0.4% paraformaldehyde; all of the incubations were performed in an incubator set to 37 °C. VCAM-1 expression on the glioblastoma cells was preliminarily confirmed by incubating them with a fluorescent anti-VCAM-1 antibody for 15 min and observing under a confocal microscope.

For the assay, NAMP and NeutrAvidin^TM^ stock aqueous solutions and the liposomal suspension in PBS were appropriately diluted with DMEM culture medium to have a total incubation volume of 300 μL. Approx. 3 × 10^4^ cells/dish were firstly incubated with 4.2 nmol of NAMP for 15 min at 37 °C; after 3 washings, 0.36 nmol of NeutrAvidin^TM^ was added; in the end, after 3 washings, 200 μL of the suspension of CM-DiI-labeled F1 or F2 liposomes was incubated with the cells for 2 or 4 h. The control cells did not receive NAMP but were only incubated with NeutrAvidin^TM^ followed by liposomes; fresh cell culture medium was added when the positive cells were treated with NAMP.

After the final incubation, the cells were washed and imaged in PBS under a confocal microscope at λ = 570 nm.

F1 and F2 liposomes were also tested on HUVECs, with a final evaluation performed using FACS. In this case, the medium used for NAMP and NeutrAvidin^TM^ stock solutions and the liposomal suspension was PBS with Ca^2+^ and Mg^2+^ to have a total incubation volume of 100 μL. Before the experiment, the cells underwent TNF-α stimulation (10 ng/mL for 4 h at 37 °C), and VCAM-1 expression was confirmed by using a phycoerythrin-labeled anti-VCAM-1 antibody; the control cells did not receive TNF-α stimulation. After TNF-α treatment, the cells were washed and transferred into Eppendorf tubes using a scraper and finally centrifuged at 1100 rpm for 8 min.

For the assay, the cells were firstly incubated with 3 nmol of NAMP, followed by 3 nmol of NeutrAvidin^TM^ and finally 100 μL of CM-DiI-labeled F1 or F2 liposomes. After each incubation step, which took place at 4 °C for 15 min, the cells were washed and centrifuged at 1100 rpm for 8 min. FACS analysis was performed by monitoring λ = 570 nm.

A further in vitro test was performed on F1a-MH liposomes. HUVECs (approx. 7 × 10^5^/dish) were stimulated with TNF-α as reported above, then underwent the treatments with DMEM solutions of 20 nmol NAMP and 20 nmol Neutravidin^TM^, and finally incubated with 800 μL of F1a-MH liposomes diluted with DMEM; 3 washings were performed after each incubation step, and all the incubations were carried out at 4 °C for 15 min. After the final incubation and washings, the cells were detached from the plate using a scraper and homogenized.

The MH cell content was measured using a gas chromatography–mass spectroscopy (GC-MS) method previously developed by our research group [34] using a Hewlett Packard 5890 Series II gas chromatograph (Agilent Technologies, Santa Clara, CA, USA) equipped with a Hewlett Packard 5971 A mass selective detector (Agilent Technologies, Santa Clara, CA, USA). Briefly, the cell homogenate was extracted with methanol, and the extract was purified via solid phase extraction using a weak cation exchanger (Strata-X-CW, 1 mL, 30 mg sorbent mass, Phenomenex, Aschaffenburg, Germany). The purified extract, with [^2^H_6_]-metformin hydrochloride added as an internal standard, was derivatized using *N*-methyl-bis(trifluoroacetamide). The analyses were performed in the selected ion monitoring mode using a cool-on-column injector at constant flow (0.5 mL/min), and the *m*/*z* = 303 and *m*/*z* = 309 ions, corresponding to derivatized MH and derivatized internal standard, respectively, were monitored.

## 3. Results

### 3.1. Unloaded Liposome Preparation, Purification and Characterization

#### 3.1.1. Liposome Preparation and Purification

Using the lipid mixtures reported in Table 1, F1 and F2 liposomes were prepared via the thin film hydration method.

The prepared liposomal formulations were purified through the use of SEC using semipreparative HPLC, and the chromatographic elution was monitored via RI and UV-DAD detectors configurated in series. The liposomes were eluted at about 5 min, while a second peak, related to ions and small molecules not included in the vesicles, was eluted at about 11–12 min.

#### 3.1.2. Liposome Size, Zeta Potential and Concentration

The size, zeta potential, and concentration values of the two empty liposomal formulations are reported in Table 2. These parameters were measured both before and after the purification procedure, and no significant changes were observed.

Liposomal stability during storage at 4 °C was investigated by monitoring the size and zeta potential: while the F1 liposomes were stable for over one year, after two weeks storage, the F2 liposomes showed statistically significant variations in average hydrodynamic diameter, while the zeta potential was quite unchanged. Therefore, it was established that F2 liposomes should be used for further studies within ten days after preparation; indeed, despite their reduced storage stability, F2 liposomes might exhibit different favorable features compared to F1 liposomes, so they were deemed worth further study.

#### 3.1.3. Qualitative Determination of Biotinylated Lipid Presence

A HABA displacement assay proved the avidin-binding ability of both liposomal formulations. When dissolved in a NeutrAvidin^TM^ solution, HABA forms a colored complex that absorbs at λ = 500 nm; the assay is based on the lower avidin-binding affinity of HABA compared to biotin (Kd = 6 × 10^−6^ M and 1.3 × 10^−15^ M, respectively); therefore, biotin and biotinylated compounds are able to displace HABA from the complex with avidin, decreasing 500 nm UV absorbance. By performing the assay on F1 and F2 formulations at the conditions reported in Section 2.5.2, an approx. 25% reduction in the 500 nm absorbance of the HABA-NeutrAvidin^TM^ complex was spectrophotometrically determined, revealing a displacement coherent with the theoretical %mol of biotinylated phospholipids included in the liposomes used to perform the test.

#### 3.1.4. TEM Analysis

**Figure 2:**
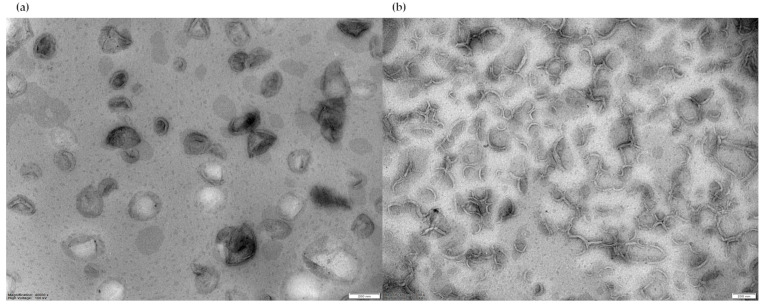
(**a**) TEM image of F1 liposomes; (**b**) TEM image of F2 liposomes. Magnification bar = 200 nm, magnification 40,000× (left), 30,000× (right), high voltage 100 kV.

For negative-stain TEM analysis, liposome suspensions in PBS with a final lipid concentration of approximately 22 mM were stained with 2% uranyl acetate, then a 20 µL sample of liposome suspension was observed. Both F1 and F2 micrographs evidenced the presence of unilamellar liposomes (Figure 2).

#### 3.1.5. ^31^P-NMR for Lamellarity Determination

To further confirm the unilamellarity of the vesicles, a ^31^P-NMR study using praseodymium salt (Pr(NO)_3_ ∙ 6H_2_O) as the shift reagent [36,37] was performed on the F1 and F2 liposome suspensions in a HEPES buffer of 10 mM and 150 mM of NaCl (pH 7.4).

**Figure 3:**
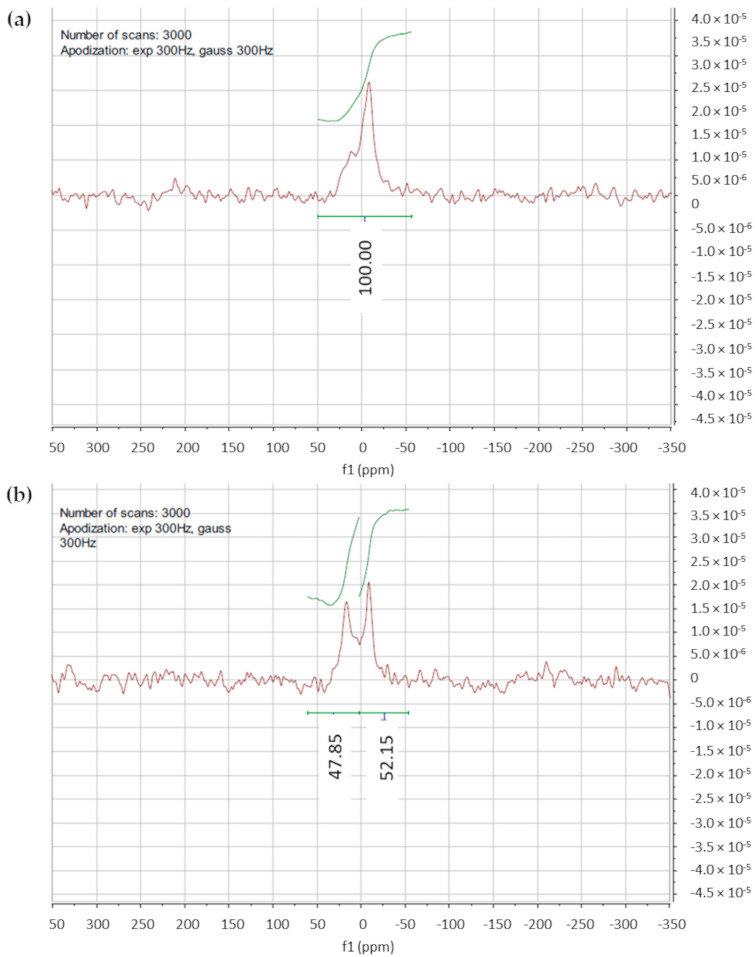
(**a**) ^31^P-NMR spectra of F1 liposomes without shift reagent; (**b**) ^31^P-NMR spectra of F1 liposomes acquired after the shift reagent addition; a partial downfield shift of the signal is evidenced.

The ^31^P-NMR spectrum of the F1 liposomes without shift reagent is shown in Figure 3a. The signal with δ = 0 ppm corresponds to phosphorous nuclei, and apodization at 3000 Hz was applied to overcome the issue of the low-intensity signal. After the addition of Pr(NO)_3_ ∙ 6H_2_O, a downfield shift in the NMR peak occurred (Figure 3b). By applying Equation (1), the results obtained for F1 and F2 liposomes, 1.04 and 1.24, respectively, indicated the unilamellarity of the vesicles.

#### 3.1.6. Cholesterol Content Determination

A rapid and accurate TLC method was developed to estimate the cholesterol content in the liposomal formulations; in particular, using a semi-automatic applier, we were able to deposit highly precise and reproducible volumes of the sample on the TLC plates, while detection was performed by means of a densitometric scanner. Normal phase chromatography was carried out using a mobile phase composed of cyclohexane/diethyl ether 3:2.5 (*v*/*v*).

**Figure 4:**
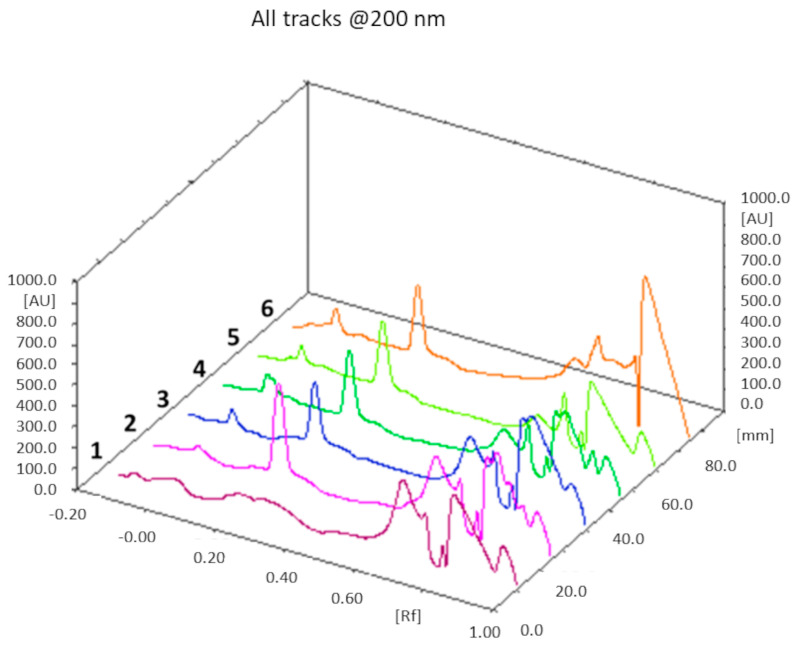
TLC chromatogram. Track 1: chloroform; track 2: mixture of cholesterol with the phospholipids of the F1 formulation dissolved in chloroform; tracks 3, 4, 5, and 6: F1 liposomes dissolved in chloroform after lyophilization. Cholesterol Rf = 0.22.

The theoretical cholesterol content was about 26% and 18% (*w*/*w*) of the total weight of all components in the F1 and F2 liposome formulations, respectively: from the TLC determination (*n* = 3), we obtained a value of 25 ± 1% (*w*/*w*) for F1 liposomes, and 17 ± 2% (*w*/*w*) for the F2 formulation. In both cases, the theoretical values of the cholesterol content fall within the experimentally found ranges. An example of a TLC chromatogram for the F1 formulation is shown in Figure 4.

### 3.2. MH-Loaded Liposome Preparation and Purification

Considering the intrinsic low stability and potential toxicity of the F2 formulation, MH loading was only performed with the F1 formulation.

MH was loaded into the F1 liposomes following the three different preparation procedures reported in Section 2.3, and the obtained MH-loaded formulations were purified through semipreparative HPLC-UV-RI.

The quantification of unencapsulated MH from the HPLC-UV-RI chromatograms could have been inaccurate since unencapsulated MH co-elutes with all small molecules, including impurities that may be present; moreover, MH that had been possibly lost during the preparation procedure could not be factored in. Therefore, a method for the direct determination of MH loaded into purified liposomes was developed, as described in the following section.

### 3.3. MH-Loaded Liposome Characterization

#### 3.3.1. MH Loading Determination

Similar to cholesterol determination, a TLC method was developed to accurately measure MH loading in the three liposomal formulations.

**Figure 5:**
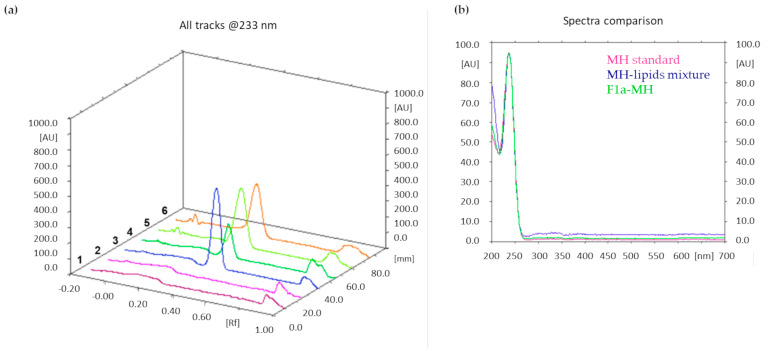
(**a**) TLC chromatogram. Track 1: methanol; track 2: mixture of lipids at the same theoretical ratio of formulation F1; track 3: 0.1 mg/mL MH standard solution; track 4: mixture of MH (0.2 mg/mL) with lipids; tracks 5 and 6: F1a-MH liposomes dissolved in methanol, after lyophilization. MH Rf = 0.35. (**b**) UV spectra extracted from the peaks with Rf = 0.35 of the chromatograms in comparison.

After lyophilization, accurately weighed amounts of the three MH-loaded liposome formulations were dissolved in methanol. The mobile phase was composed of ammonium sulfate (0.025 M)/acetonitrile 7:3 (*v*/*v*); absorbance at λ = 233 nm was monitored. Additionally, in this case, the MH content was quantified by integrating the peak area of the metformin band after migration. Figure 5a shows an illustrative chromatogram; the UV spectra extraction from the chromatogram confirmed that the peaks in tracks 5 and 6, having the same Rf as the MH standard solution, corresponded to MH (Figure 5b).

The drug loading results obtained from F1a-MH, F1b-MH, and F1c-MH were 3.2 ± 0.3, 2.6 ± 0.2, and 1.5 ± 0.2, respectively.

ANOVA analysis, as reported in Table S1, was performed to evaluate the statistical differences among the three MH-loaded formulations in terms of drug loading capacity. The three drug loading values deriving from the three MH-encapsulating procedures were significantly different, as evidenced by the *p*-value, which is <0.001; therefore, the statistical analysis evidenced that the three loading techniques exhibited different efficiencies (as clearly appreciable in Figure S1); in particular, the most successful involved lipid film hydration with an MH aqueous solution.

The significant differences among all of the formulations were also confirmed through the use of the post hoc tests (Tukey’s, Bonferroni, and Fisher’s Test) performed to compare the formulations in pairs (Table S2).

Additionally, the encapsulation efficiencies of the three MH-loaded formulations were determined: 4.8 ± 0.5% for F1a-MH liposomes, 3.4 ± 0.6% for F1b-MH liposomes, and 2.1 ± 0.5% for F1c-MH liposomes. These relatively low encapsulation efficiency values might be explained by considering the high hydrophilicity of MH, and future studies will address improving the encapsulation efficiency by optimizing the volume and concentration of the MH solution used within the loading procedure. Nevertheless, it has to be taken into account that, in sight of good drug loading results, low encapsulation efficiency values can be considered acceptable for non-expensive drugs, like MH.

In conclusion, the F1a-MH formulation, featuring the highest drug loading, was the best formulation among the MH-loaded liposomes.

#### 3.3.2. Loaded Liposome Size and Zeta Potential

The size, PDI and zeta potential values of the MH-loaded formulations are reported in Table 3. The mean hydrodynamic diameter and zeta potential of the MH-loaded formulations were not significantly different in terms of the mean size and zeta potential of empty F1 liposomes (*p* > 0.05).

To evaluate the differences in terms of size and zeta potential of the F1 formulation and the three MH-encapsulating formulations, ANOVA analysis was carried out.

The analysis of variance results obtained by comparing the mean size values of the four formulations are shown in Table S3: the four populations were significantly different, as highlighted by the *p*-value of < 0.05. Three different post hoc tests (Tukey’s, Bonferroni, and Fisher’s Test) were performed to compare the formulations in pairs (Table S4): in particular, all of the post hoc tests evidenced that F1, F1a-MH, and F1b-MH were not significantly different from each other, while F1c-MH was significantly different from F1a-MH and F1b-MH, as highlighted by the *p* value of < 0.05; furthermore, the Fisher’s post hoc test also showed a significant difference between F1 and F1c-MH, which was not highlighted by the other two post hoc tests. The mean size values of the four populations are graphically represented in Figure S2. To explain the size increase in F1c-MH liposomes, it has to be considered that, in this formulation, the thin lipid film was formed in the presence of MH, which might remain associated with the external liposome surface, with a consequent enlargement in vesicle hydrodynamic diameter. Instead, a mean size increase may not occur in F1b-MH liposomes because, in this case, MH methanolic solution was added after lipid film formation, and under these conditions, MH might stratify the lipids film, preventing MH association to the exterior of the liposomes.

Table S5 shows the outputs of the analysis of variance comparing the zeta potential values: in this case, no significant difference among the formulations was evidenced, with *p* values of >0.05. This result was confirmed via Tukey’s and Bonferroni post hoc tests, while Fisher’s test highlighted a significant difference between the F1a-MH and F1b-MH zeta potential values (Table S6). The graphical representation of the zeta potential values (Figure S3) clearly shows that the F1a-MH liposomes retain a zeta potential value similar to F1 liposomes, while F1b-MH and F1c-MH exhibit higher zeta potentials. In fact, the preparation procedures of F1b-MH and F1c-MH are similar while being clearly different from the one used to prepare the F1a-MH formulation: during the formation of F1b-MH and F1c-MH liposomes, MH might electrostatically associate to the outer layer of the liposomes, influencing their zeta potential. Several studies reporting that MH acts at the cell membrane level by increasing membrane fluidity [38,39] support the hypothesis of MH interaction with liposome membranes.

After two weeks of storage at 4 °C, the three MH-loaded formulations did not show significant changes in mean size and zeta potential (*p* > 0.05) compared to freshly prepared formulations.

Considering the optimal results obtained with the F1a-MH formulation in terms of drug loading and stability while retaining mean size and zeta potential values similar to empty F1 liposomes, this MH-loaded formulation was selected for in vitro tests on cells.

### 3.4. In Vitro Tests

Preliminary in vitro tests were performed on two cell lines to confirm the feasibility of the three-step protocol.

A preliminary confocal microscopy test was performed on glioblastoma cells, naturally overexpressing the adhesion protein VCAM-1, after verifying the VCAM-1 expression levels by using a phycoerythrin-labeled anti-VCAM-1 antibody. Approx. 3 × 10^4^ cells/dish were first incubated with NAMP for 15 min; then, after washings, NeutrAvidin^TM^ was added; finally, after repeating the washings, CM-DiI-labeled F1 or F2 liposomes were added, followed by incubation for 2 or 4 h. Control cells only received NeutrAvidin^TM^ and the CM-DiI-labeled liposomes.

**Figure 6:**
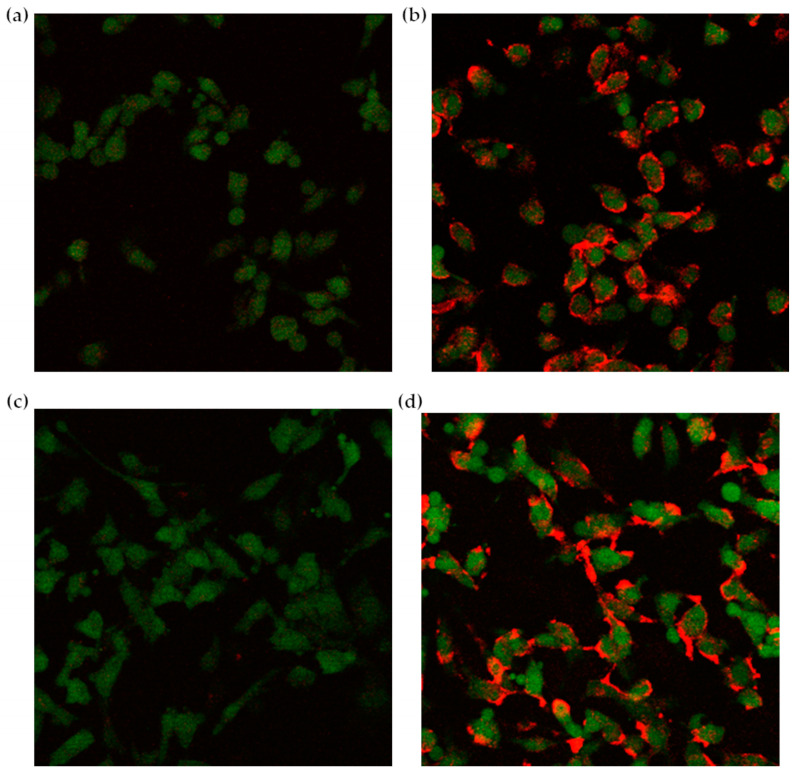
Confocal microscopy images of glioblastoma cells. (**a**,**c**): control cells receiving only NeutrAvidin^TM^ and the biotinylated liposomes; (**b**,**d**): cells treated with the complete three-step protocol (NAMP-NeutrAvidin^TM^-biotinylated liposomes); a 2 h incubation time in (**a**,**b**), and a 4 h incubation time in (**c**,**d**).

Confocal images highlighted a preferential interaction of fluorescently labeled liposomes with cells treated with the complete three-step protocol (Figure 6). Due to the stable GFP expression induced by retroviral infection, glioblastoma cells appear green under the confocal microscope, while the red CM-DiI signal, deriving from the labeled liposomes, is clearly more intense in cells first treated with NAMP, followed by NeutrAvidin^TM^, and finally, biotinylated liposomes (Figure 6b,d). On the contrary, cells incubated with only NeutrAvidin^TM^ followed by biotinylated liposomes show negligible red signal (Figure 6a,c). This finding evidenced that NeutrAvidin^TM^ and biotinylated liposomes did not interact specifically with glioblastoma cells, while biotinylated liposomes noticeably interacted with cells previously receiving both NAMP and NeutrAvidin^TM^. Interestingly, we can also observe that some red “clusters” are formed after 4 h incubation with liposomes (Figure 6d); these aggregates might be due to the binding of more than one biotinylated liposome to NeutrAvidin^TM^. Nevertheless, NeutrAvidin^TM^ is endowed with four biotin-binding sites, and longer incubation time might favor the interaction of more than one biotinylated liposome (up to three, in theory) with a single NeutrAvidin^TM^ molecule bound to NAMP and, since the cells were fixed prior to the experiment, no internalization of liposomes could occur.

Further in vitro tests were performed on HUVECs via FACS analysis. VCAM-1 expression was induced in positive cells via stimulation with 10 ng/mL TNF-α for 4 h; the concentration and incubation time were suitably optimized in order to induce VCAM-1 expression without compromising cell integrity. Additionally, in this case, VCAM-1 expression was controlled before carrying out the experiments by using a phycoerythrin-labeled anti-VCAM-1-antibody.

**Figure 7:**
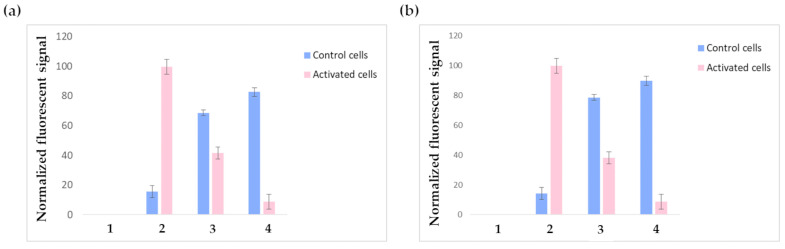
FACS results obtained by testing F1 (**a**) and F2 (**b**) liposomes. Non-activated cells are represented by blue bars, while pink bars represent TNF-α-activated cells. 1: negative controls (plain cells); 2: positive controls (cells receiving anti-VCAM-1 antibody); 3: cells treated with only NeutrAvidin^TM^ followed by F1 or F2 liposomes; 4: cells treated with the complete three-step pretargeting protocol: first NAMP, followed by NeutrAvidin^TM^ and F1 or F2 liposomes.

Figure 7a shows the outputs of the FACS experiments performed on HUVECs receiving the three-step pretargeting treatment using F1-labeled liposomes; the results are normalized on the fluorescent intensity signal of the positive control cells incubated with phycoerytrin-labeled anti-VCAM-1-antibody (pink bar in 2). As expected, the constitutive expression of VCAM-1 was negligible in non-stimulated cells (blue bar in 2), while after TNF-α stimulation, VCAM-1 expression was consistently increased. Contrary to glioblastoma cells, TNF-α stimulated HUVECs treated with just NeutrAvidin^TM^ and the biotinylated liposomes show significant aspecific binding. It must be considered that even if the TNF-α stimulation conditions have been suitably optimized to induce VCAM-1 expression without compromising cell integrity, TNF-α stimulation still remains a stress factor for cells, which undergo alterations regarding their metabolism and the expression levels of several receptors; indeed, since TNF-α is a pleiotropic cytokine, it might modify not only cell adhesion molecules but also other endogenous biotin-binding proteins, which can cause background signals. These altered conditions might explain the observed aspecific binding. The fluorescent signal decrease in TNF-α-activated HUVECs receiving the complete three-step treatment (bar 4) compared to the controls might be explained by considering possible liposome internalization. Indeed, previous studies reported that liposomes, after entering the cell, are rapidly degraded in just 15–20 min [40]. It also has to be considered that carbocyanine dyes, like CM-DiI, exhibit weak fluorescence in water while becoming fluorescent when included in membranes; this might explain the loss in fluorescent signal. Moreover, it is known that TNF-α activates endothelial cells by inducing the synthesis of numerous angiogenic factors, the expression of increased levels of adhesion molecules, and the generation of reactive oxygen species; these changes are accompanied by increased oxygen consumption and mitochondria biogenesis [41,42]. Such an activated state might reasonably induce increased cellular transport rates and boosted metabolism in endothelial cells, leading to the accelerated degradation of endocytosed compounds. Anyway, this result was not in contrast with the confocal microscopy experiments performed on glioblastoma cells, since glioblastoma cells were fixed before the experiments, so all possible internalization phenomena were prevented. Future tests will be conducted by replacing CM-DiI with a different fluorescent dye.

By testing F2 liposomes under the same conditions described for F1 liposomes, similar results were obtained (Figure 7b); nevertheless, F2 liposomes displayed higher toxicity: indeed, all experiments were carried out using the same initial number of cells but, in the experiments involving F2 liposomes, the FACS elaboration recognized as dead a higher number of cells (these cells were not considered during data elaboration). This observation is in accordance with the well-known cytotoxicity of positively charged liposomes.

F1a-MH liposomes, featuring the best MH-loading among the studied formulations, were used in a further in vitro study on HUVECs. Additionally, in this case, VCAM-1 expression was stimulated by treatment with TNF-α and, after incubation with NAMP, followed sequentially by NeutrAvidin^TM^ and F1a-MH liposomes, the cells were detached from the culture plates and homogenized.

The cell homogenates underwent the extraction, purification, and derivatization procedure set up in our previous work, and the obtained derivatized solutions were analyzed through the use of GC-MS. As a control, the cells that had not been previously activated with TNF-α underwent the same treatment.

Our previously developed GC-MS method, exhibiting a linearity ranging from 0.24 to 4.12 μM and a sensitivity of 0.37 μM, allowed us to detect the presence of MH in VCAM-1 overexpressing HUVECs: approx. an average of 3.78 ± 0.09 pmol (*n* = 3) was overall internalized by the cultured cells, while the MH content in the control cells was lower than the LOD of the method (corresponding to approx. 0.37 pmol MH). This result corroborates the hypothesis of the rapid internalization and decomposition of the liposomes into the VCAM-1 overexpressing cells.

## 4. Discussion

Two stealth liposomal formulations with different zeta potentials determined by their different lipid compositions were prepared using thin film hydration. The F1 formulation was intended to be negatively charged and, besides DSPE-PEG_2000_-biotin, also included DSPE-PEG_2000_. The total content of PEGylated phospholipids was maintained in a molar percentage of about 3 mol%, a concentration normally employed in stealth formulations, definitely below the limit value of 7 mol%; indeed, above this value, PEG chains are subjected to a transition from mushroom to brush conformation, which is known to produce micelle formation and consequent instability phenomena [43,44].

With the F2 formulation, we also tested cationic liposomes. A positive charge might favor the interaction of liposomes with cell membranes, but it is also well known that positively charged particles may interact with different enzymes, like protein kinase C, and with other components of cell membranes, causing toxicity [45].

The lipid film hydration temperature was set considering the glass transition temperature (Tg) of the lipids included in each formulation; therefore, since the F1 formulation included DSPC exhibiting a Tg = 55 °C, the hydration and extrusion processes took place at 58 ± 1 °C, while the F2 formulation was hydrated at 46 ± 1 °C since DMDAB exhibits a Tg of about 43 °C [46].

An efficient SEC purification, exploiting a semipreparative HPLC system, was performed before liposome characterization.

A key aspect of the characterization of a nanoformulation that is intended to be employed in pharmaceutical applications is the determination of the concentration; therefore, we determined the concentration of our formulation through the use of NTA. Indeed, by determining the vesicle concentration, it is possible to formulate related risk assessments, implement effective quality assurance and quality control protocols, and ensure compliance with present and future regulations, such as the EC Recommendation for the definition of a nanomaterial (2011/696/EU) [47].

F1 liposomes were stable for over one year under storage conditions, while F2 liposomes showed higher instability. The low stability of F2 liposomes could be explained by considering the very low Tg (−20 °C) of the phospholipid DOPC that might form liposomes with high phospholipid layer fluidity, possibly leading to fusing phenomena during storage. Moreover, the C18 unsaturated chains of DOPC easily undergo degradation due to oxidation.

Both F1 and F2 formulations demonstrated avidin-binding ability, confirming the presence of the biotin moiety on the external layer of the phospholipid bilayer, and the unilamellarity of the obtained vesicles was verified via both visual observation, through TEM analysis, and ^31^P-NMR. The liposomal cholesterol content, determined for both formulations, was coherent with the theoretical value.

All of the physico-chemical features of the described liposomal formulations, including size, zeta potential, and morphology, might influence their pharmacokinetics; ongoing studies are focused on the evaluation of these important aspects.

MH, a potential anti-inflammatory and anticancer agent, was loaded in F1 liposomes by following three different procedures. F1a-MH liposomes were prepared by using the classic passive loading procedure, which is typically employed when encapsulating hydrophilic drugs; indeed, an MH aqueous solution was used to hydrate the lipid film. In F1b-MH liposomes, MH in methanolic solution was added after the formation of the lipid film, while in F1c-MH liposomes, MH in methanolic solution was added to the lipid mixture in chloroform before the formation of the thin film. In the latter formulations, the aim was to include MH in the lipid film to increase the drug entrapment into the vesicles [48]. In particular, the three MH-loaded formulations were compared for their size, zeta potential, and drug loading values. The size increase in F1c-MH liposomes might be due to an MH association with the exterior of liposomes with a consequent enlargement in vesicle diameter, since, in this formulation, the thin lipid film was formed in the presence of MH. Contrarily, in F1b-MH liposomes, the MH methanolic solution was added after the lipid film formation, reducing MH association to the exterior of the liposomes and preventing a mean size increase. F1c-MH also exhibited increased zeta potential values, corroborating the hypothesis concerning MH association with the surface of the liposomes.

Considering the drug loading values obtained, formulation F1a-MH was the most promising.

The presented three-step pretargeting system is based on the strong and well-known avidin–biotin interaction: avidin is a homotetrameric protein of about 68 kDa, obtained from egg whites, with each subunit presenting a biotin-binding site. Avidin is highly glycosylated and exhibits basics features, with an isoelectric point around 10 [49,50]. Streptavidin, produced by *Streptomyces avidinii*, is structurally similar to avidin, although low sequence homology characterizes the two proteins. Streptavidin lacks glycosylation and, compared to avidin, has lower molecular mass (about 56 kDa), net charge, and immune reactivity [51]. Moreover, streptavidin exhibits a lower isoelectric point (about 7) due to the lower number of lysine and arginine residues. Avidin has been long exploited for immunohistochemistry applications, but a high degree of non-specific interaction, mainly due to lectin binding, has been evidenced; contrarily, streptavidin displays reduced non-specific binding thanks to the lack of carbohydrate chains and the lower isoelectric point [52]. Nevertheless, because of the RYD motif (a bacterial recognition sequence similar to the mammalian RGD motif) contained in the streptavidin sequence, some assays employing streptavidin were affected by background signal. NeutrAvidin^TM^, obtained from deglycosylation of avidin, displays a low isoelectric point (about 6.3), reduced non-specific interactions, and low production cost. Therefore, for the preliminary in vitro tests, NeutrAvidin^TM^ was selected as the biotin-binding molecule employed in the second step of the pretargeting protocol.

An important aspect to be considered while performing the in vitro tests was the presence of divalent cations in the incubation medium since these cations might determine the successful binding of NAMP to VCAM-1. Indeed, the NAMP molecule includes the VHPKQHR amino acid sequence, homologous of the α subunit of integrin α_4_β_1_, one of the main natural VCAM-1 ligands; several studies report the important influence of divalent cations, like Mg^2+^ and Mn^2+^, on the strength of α_4_β_1_-VCAM-1 binding [53,54,55]. Therefore, DMEM cell culture medium (containing Ca^2+^ and Mg^2+^) or PBS added with Ca^2+^ and Mg^2+^ was used as the incubation media during the in vitro tests.

Confocal microscopy evidenced a high and specific interaction between fluorescent biotinylated liposomes and glioblastoma cells, overexpressing VCAM-1 after receiving the complete three-step pretargeting treatment. FACS analysis, performed on HUVECs, revealed a decreased fluorescent signal deriving from TNF-α-activated cells, compared to non-stimulated control cells: this result might be explained by considering a possible rapid internalization and degradation of the fluorescently labeled liposomes. This hypothesis was corroborated by the results obtained by treating TNF-α-stimulated HUVECs with F1a-MH liposomes, evidencing a high content of MH in the activated cells receiving the complete three-step pretargeting treatment.

The obtained results certainly deserve further in-depth studies, and the hypothesis of rapid internalization and metabolism of liposomes in the VCAM-1-overexpressing cells should be confirmed via live confocal microscopy experiments. Indeed, in our previous work describing the use of this pretargeting system employing a biotinylated radiotracer as the third step of the pretargeting protocol, the signal deriving from the TNF-α stimulated HUVECs evidenced a continuous radioactive uptake over 30 min without significant decrease. However, the cellular metabolism of the biotinylated liposomes might be faster than the metabolism of the biotinylated radioligand used in previous experiments. Moreover, the possibility of internalization of the VCAM-1-NAMP–avidin–radiotracer complex was not investigated in our previous work.

## 5. Conclusions

We developed a three-step pretargeting system based on avidin–biotin interaction for diagnostic and therapeutic applications in pathologies characterized by the overexpression of the VCAM-1 adhesion protein. Indeed, in our previous work, we described the synthesis and characterization of a VCAM-1-binding molecule named NAMP. In our previous work, the diagnostic feasibility of this three-step pretargeting approach was demonstrated, while, in the present work, we aimed to demonstrate the therapeutic applicability of the system by describing the development of a nanocarrier exploitable for the vehiculation of anti-inflammatory drugs. Indeed, even if pretargeting has been initially conceived for imaging purposes, these kinds of strategies are under investigation for their possible application in the therapeutic field, and the new pretargeting system here proposed, based on NAMP, might represent a flexible theranostic tool exploitable for both diagnoses, by using a biotinylated radiotracer, and therapy, by using a biotinylated nanocarrier encapsulating an active compound with anti-inflammatory activity.

Two different biotinylated liposome formulations, an anionic and a cationic one, were prepared and fully characterized; the anionic one appeared more promising due to its long-term stability and absence of intrinsic toxicity on the cell lines used for testing, so it was exploited for preliminary studies on metformin encapsulation, loaded via three different procedures.

Confocal microscopy observations and FACS analysis performed on glioblastoma cells and HUVECs proved the efficacy of the three-step pretargeting approach, supporting the potential therapeutic feasibility of the system. Moreover, the three-step procedure involving the use of MH-loaded liposomes revealed MH accumulation in cells.

Future studies employing liposomes encapsulating other anti-inflammatory or anticancer actives will be performed to confirm the therapeutic efficacy of the targeted strategy. Moreover, ongoing studies are focused on evaluating the possibility of the preliminary formation of the NAMP–NeutrAvidin^TM^ complex in vitro, which might be used in a two-step pretargeting system before the injection of drug-loaded biotinylated liposomes: this procedure might have the advantage of two injections instead of three and prevent any steric issues that NeutrAvidin^TM^ might encounter when interacting with the biotinylated moiety of a NAMP molecule already bound to VCAM-1.

## Figures and Tables

**Table 1:** Composition of the phospholipid mixtures used to obtain the F1 (anionic) and F2 (cationic) liposomal formulations.

Formulation	DSPC (mol%)	DOPC (mol%)	DMDAB (mol%)	Cholesterol (mol%)	DSPE-PEG_2000_ (mol%)	DSPE-PEG_2000_-Biotin (mol%)
F1	51.8			45.0	3.0	0.2
F2		60.0	10.0	29.8		0.2

**Table 2:** Size, PDI, zeta potential, and concentration of F1 and F2 liposomes. Size, zeta potential, and concentration are expressed as mean ± standard deviation (*n* = 20).

Formulation	Size (nm)	PDI	Zeta Potential (mV)	Concentration (Particles/mL)
F1	164 ± 15	0.04–0.10	−16 ± 2	1.2 ± 0.1 × 10^13^
F2	179 ± 30	0.21–0.27	+20 ± 2	1.3 ± 0.1 × 10^14^

**Table 3:** Size, PDI, and zeta potential of the MH-loaded liposomes. Size and zeta potential are expressed as mean ± standard deviation (*n* = 3).

Formulation	Size (nm)	PDI	Zeta Potential (mV)
F1a-MH	163 ± 7	0.03–0.09	−17 ± 1
F1b-MH	160 ± 7	0.06–0.07	−13 ± 3
F1c-MH	185 ± 8	0.04–0.08	−14 ± 1

## Data Availability

The original contributions presented in the study are included in the article/supplementary material, further inquiries can be directed to the corresponding author.

## Supplementary Materials

The following supporting information can be downloaded at: https://www.mdpi.com/article/10.3390/pharmaceutics16020235/s1, Table S1: ANOVA comparing the drug loading values of F1a-MH, F1b-MH and F1c-MH formulations; Figure S1: drug loading values of the three MH-loaded liposomal formulations; Table S2: post hoc analyses applied to the ANOVA performed on the drug loading data; Table S3: ANOVA comparing the mean size of unloaded and MH-loaded F1 liposomes; Table S4: post hoc analyses applied to the ANOVA performed on the mean size data of unloaded and MH-loaded F1 liposomes; Figure S2: mean size values of unloaded and MH-loaded F1 liposomes; Table S5: ANOVA comparing the mean zeta potential values of unloaded and MH-loaded F1 formulations; Table S6: post hoc analyses applied to the ANOVA performed on the zeta potential values of unloaded and MH-loaded F1 liposomes; Figure S3: mean zeta potential values of unloaded and MH-loaded F1 liposomes.
