# Supplementary material for: Development of Biotinylated Liposomes Encapsulating Metformin for Therapeutic Targeting of Inflammation-Based Diseases
Source: Pharmaceutics. 2024 Feb 5;16(2):235. doi: 10.3390/pharmaceutics16020235 (PMC10893420; doi:10.3390/pharmaceutics16020235)
Supplement: Supplementary file 1 [file pharmaceutics-16-00235-s001.zip › pharmaceutics-2822917-supplementary.pdf]

Supplementary Materials

Table S1. Analysis of variance performed to compare the drug loading values of F1a-MH, F1b-MH and F1c-MH formulations.

ANALYSIS OF VARIANCE

Source	Type III SS	df	Mean Squares	F-ratio	p value
**VAR$(1)**	6.585	2	3.293	68.354	0.000
**Error**	0.434	9	0.048		

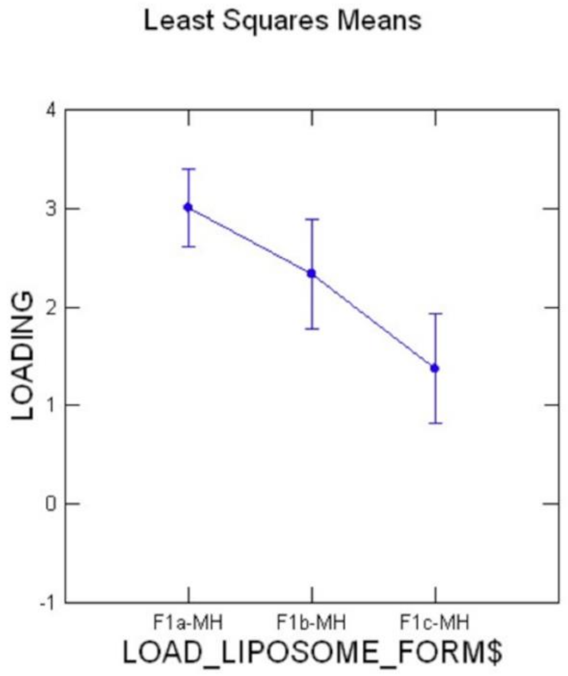

Figure S1. Drug loading values of the three MH-loaded liposomal formulations, in a graphical representation.

Table S2. *Post-hoc* analyses results deriving from the Tukey's (A), Bonferroni (B) and Fisher's (C) tests, applied to the ANOVA performed on the drug loading data.

A

Tukey's Honestly-Significant-Difference Test					
VAR$(1)(i)	VAR$(1)(j)	Difference	p-Value	95% Confidence Interval	
				Lower	Upper
F1a metf	F1b metf	0,671	0,005	0,238	1,105
F1a metf	F1c metf	1,796	0,000	1,362	2,229
F1b metf	F1c metf	1,124	0,000	0,691	1,558

B

Bonferroni Test					
VAR$(1)(i)	VAR$(1)(j)	Difference	p-Value	95% Confidence Interval	
				Lower	Upper
F1a metf	F1b metf	0,671	0,006	0,216	1,126
F1a metf	F1c metf	1,796	0,000	1,340	2,251
F1b metf	F1c metf	1,124	0,000	0,669	1,580

C

Fisher's Least-Significant-Difference Test					
VAR$(1)(i)	VAR$(1)(j)	Difference	p-Value	95% Confidence Interval	
				Lower	Upper
F1a metf	F1b metf	0,671	0,002	0,320	1,022
F1a metf	F1c metf	1,796	0,000	1,445	2,147
F1b metf	F1c metf	1,124	0,000	0,773	1,476

Table S3. Analysis of variance comparing the mean hydrodynamic diameter of unloaded and MH-loaded F1 formulations.

ANALYSIS OF VARIANCE

Source	Type III SS	df	Mean Squares	F-ratio	p value
LIPOSOME_FORM$	1172,595	3	390,865	4,765	0,021
Error	984,339	12	82,028		

Table S4. *Post-hoc* analyses results deriving from the Tukey's (A), Bonferroni (B) and Fisher's (C) tests, applied to the ANOVA performed on the mean size data of unloaded and MH-loaded F1 liposomes.

A

Tukey's Honestly-Significant-Difference Test					
LIPOSOME_FORM$(-i)	LIPOSOME_FORM$(-j)	Difference	p-Value	95% Confidence Interval	
				Lower	Upper
F1	F1a-MH	4,817	0,842	-12,540	22,174
F1	F1b-MH	6,733	0,767	-13,804	27,270
F1	F1c-MH	-17,300	0,110	-37,837	3,237
F1a-MH	F1b-MH	1,917	0,990	-17,097	20,930
F1a-MH	F1c-MH	-22,117	0,021	-41,130	-3,103
F1b-MH	F1c-MH	-24,033	0,031	-45,988	-2,078

B

Bonferroni Test					
LIPOSOME_FORM$(-i)	LIPOSOME_FORM$(-j)	Difference	p-Value	95% Confidence Interval	
				Lower	Upper
F1	F1a-MH	4,817	1,000	-13,615	23,248
F1	F1b-MH	6,733	1,000	-15,075	28,542
F1	F1c-MH	-17,300	0,167	-39,108	4,508
F1a-MH	F1b-MH	1,917	1,000	-18,274	22,107
F1a-MH	F1c-MH	-22,117	0,029	-42,307	-1,926
F1b-MH	F1c-MH	-24,033	0,042	-47,347	-0,719

C

Fisher's Least-Significant-Difference Test					
LIPOSOME_FORM$(-i)	LIPOSOME_FORM$(-j)	Difference	p-Value	95% Confidence Interval	
				Lower	Upper
F1	F1a-MH	4,817	0,426	-7,921	17,555
F1	F1b-MH	6,733	0,350	-8,338	21,805
F1	F1c-MH	-17,300	0,028	-32,372	-2,228
F1a-MH	F1b-MH	1,917	0,770	-12,037	15,870
F1a-MH	F1c-MH	-22,117	0,005	-36,070	-8,163
F1b-MH	F1c-MH	-24,033	0,007	-40,146	-7,921

Figure S2. Mean size values of unloaded and MH-loaded F1 liposomes.

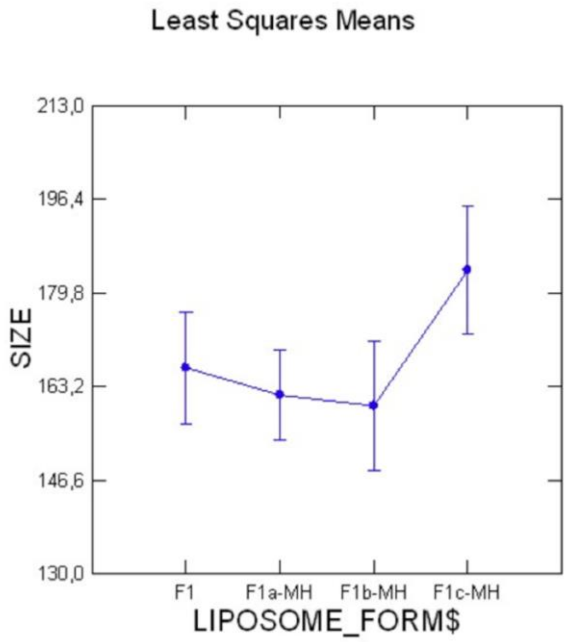

Table S5. Analysis of variance performed to compare the mean zeta potential values of unloaded and MH-loaded F1 formulations.

ANALYSIS OF VARIANCE

Source	Type III SS	df	Mean Squares	F-ratio	p value
**LIPOSOME_FZ$**	26,583	3	8,861	2,817	0,094
**Error**	31,460	10	3,146		

Table S6. *Post-hoc* analyses results deriving from the Tukey's (A), Bonferroni (B) and Fisher's (C) tests, applied to the ANOVA performed on the mean zeta potential values of unloaded and MH-loaded F1 liposomes.

A

Tukey's Honestly-Significant-Difference Test

LIPOSOME_FZ$(i)	LIPOSOME_FZ$(j)	Difference	p-Value	95% Confidence Interval	
				Lower	Upper
F1	F1a-MH	0,994	0,867	-2,968	4,957
F1	F1b-MH	-2,672	0,229	-6,635	1,291
F1	F1c-MH	-1,839	0,516	-5,802	2,124
F1a-MH	F1b-MH	-3,667	0,114	-8,097	0,764
F1a-MH	F1c-MH	-2,833	0,266	-7,264	1,597
F1b-MH	F1c-MH	0,833	0,937	-3,597	5,264

B

Bonferroni Test

LIPOSOME_FZ$(i)	LIPOSOME_FZ$(j)	Difference	p-Value	95% Confidence Interval	
				Lower	Upper
F1	F1a-MH	0,994	1,000	-3,250	5,239
F1	F1b-MH	-2,672	0,396	-6,917	1,572
F1	F1c-MH	-1,839	1,000	-6,083	2,406
F1a-MH	F1b-MH	-3,667	0,179	-8,412	1,079
F1a-MH	F1c-MH	-2,833	0,473	-7,579	1,912
F1b-MH	F1c-MH	0,833	1,000	-3,912	5,579

C

Fisher's Least-Significant-Difference Test

LIPOSOME_FZ$(i)	LIPOSOME_FZ$(j)	Difference	p-Value	95% Confidence Interval	
				Lower	Upper
F1	F1a-MH	0,994	0,460	-1,892	3,881
F1	F1b-MH	-2,672	0,066	-5,558	0,214
F1	F1c-MH	-1,839	0,186	-4,725	1,047
F1a-MH	F1b-MH	-3,667	0,030	-6,894	-0,440
F1a-MH	F1c-MH	-2,833	0,079	-6,060	0,394
F1b-MH	F1c-MH	0,833	0,578	-2,394	4,060

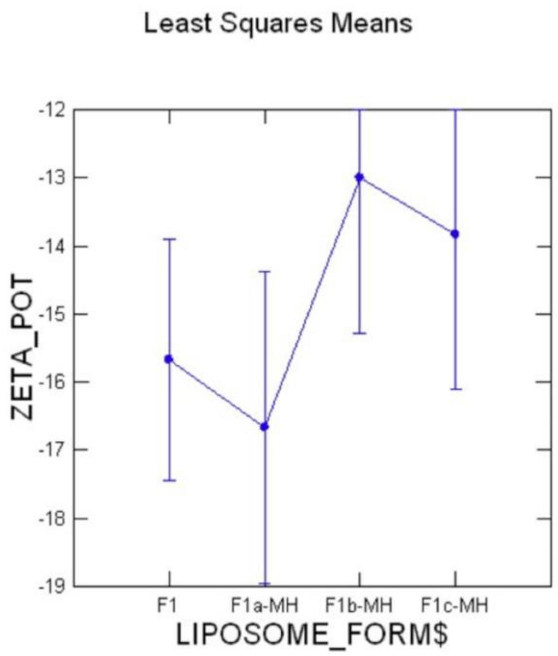

Figure S3. Mean zeta potential values of unloaded and MH-loaded F1 liposomes.
